# Use of Lead Isotopic Ratios as Geographical Tracer for Lambrusco PDO Wines

**DOI:** 10.3390/molecules25071641

**Published:** 2020-04-02

**Authors:** Lisa Lancellotti, Simona Sighinolfi, Andrea Marchetti, Lorenzo Tassi

**Affiliations:** Department of Chemical and Geological Sciences, University of Modena and Reggio Emilia, 41125 Modena, Italy; lisa.lancellotti@unimore.it (L.L.); simona.sighinolfi@unimore.it (S.S.); lorenzo.tassi@unimore.it (L.T.)

**Keywords:** wine, traceability, lead isotope ratios, MC-ICP/MS

## Abstract

In this study, the lead isotope signature was tested with the aim to verify its potential as geographic tracer for wine production and particularly for the Lambrusco PDO wines of the province of Modena (Italy). A solid phase extraction procedure, for separating lead from the investigated matrices, soil and wine, was optimized. Furthermore, different mathematical models, based on an exponential law and internal or external correction approach, were evaluated for the correction of instrumental mass dependent fractionation. The optimized analytical procedure yielded isotopic ratio data relative to the lead NIST 981 standard, ^208^Pb/^206^Pb = 2.16664 and ^207^Pb/^206^Pb = 0.914645, in good agreement both with the tabulated values and with the most recent literature data. Measured isotope ratio data highlight the contribute of multiple lead sources in bottled wine but different from the one present in soils.

## 1. Introduction

Nowadays, it is generally assumed by consumers that the highest quality of food is increasingly associated with their territorial origin. Although, the system of geographical indications, GI, namely: PDO (Protected Designation of Origin), PGI (Protected Geographical Identification) and TSG (Traditional Specialty Guaranteed) and their logos [1,2,3] is designed to facilitate and ensure the intellectual property protection of agricultural products, the characteristics that consumers identify in these logos go beyond the mere aspect of legal protection. In fact, they recognize in these denominations subjective "gustatory" requisites, associated with the "quality" of the product/process in a broader sense.

Based on the above considerations, it is clear that an objective approach to trace the origin of food and, eventually, their relations with the local environment can certainly be of valuable support to the traditional paper-based traceability systems.

This could be achieved by means of objective analytical criteria able to define the peculiar characteristics of the various products, with particular reference to their origin, in a univocal way [4].

The current objective approaches are based on analytical parameters that can be classified into direct and indirect geographical indicators [5,6]. In the case of direct indicators, among the variables able to correlate chemical characteristics of both the food and the territory of origin, the isotope ratios of stable light elements (H, C, N, O, and S) and heavy elements (Sr, Nd, and Pb) have been successfully used as tracers [7]. Despite both light and heavy isotopes are almost able to establish a direct link between the geographic area, in which the plant’s life cycle is active, and the finished product, the use of the latter ones offers several advantages. In particular, heavy elements do not show a significant fractionation during the plant uptake process making possible to develop stable models over time [8]. Recently, there has been renewed interest in the descriptive capacity of strontium demonstrating the potentials of this isotopic signature in the agri-food field [9].

However, in order to make geographic and food-chain traceability models more specific, it is often necessary to combine different indicators. In addition to Sr, lead may represent an interesting tool thanks to its peculiarities [10].

Lead is characterized by a complex isotopic pattern. In particular, it has four stable isotopes: ^204^Pb, ^206^Pb, ^207^Pb and ^208^Pb. In the Earth’s crust, the four isotopes have, approximately, the following relative abundances: ^204^Pb = 1.4%, ^206^Pb = 24.1%, ^207^Pb = 22.1%, and ^208^Pb = 52.4%. The ^204^Pb is not a radiogenic nuclide, while the others are produced by the radioactive decay of Uranium, (^235^U/^207^Pb, ^238^U/^206^Pb), and Thorium, (^232^Th/^208^Pb) [10]. Due to its low natural abundance, the analytical determination of ^204^Pb isotope produces values characterized by high uncertainty. Consequently, in order to express data as isotope ratios (IRs), the use of the following couples: ^206^Pb/^207^Pb, ^208^Pb/^206^Pb, and ^208^Pb/^207^Pb is preferred. In this way, the abundance of the ^207^Pb isotope—which has changed in a more limited way than ^206^Pb—is exploited, because the ^235^U isotope is now almost completely decayed, while the ^238^U is still present on Earth [10].

The Pb isotopic composition allows the characterization and identification of the different lead sources in natural systems, and therefore enables a reliable description of their mixing degree when all the end members are known. In fact, when both the ratios are plotted each other and a linear trend is obtained, it is possible to assume the existence of a multiple contribution of different end members. On the other hand, the natural contribution of the lead content found today in nature is minimal, since most of this element is a consequence of anthropogenic activities mainly due to a widespread use of lead in the past which generated an artificial cycle of this metal in the environment [11].

In order to evaluate the contamination sources, the mere knowledge of the total Pb concentration is not enough, while the IRs may be useful as a fingerprint of the element source, including the plant uptake by roots, the use of compounds containing lead such as fertilizers and pesticides, and the contamination due to environmental pollution [11,12].

In this scenario, the atmosphere was the former major recipient for lead emission source while, in the actual situation, soil appears to be the most important depositary of anthropogenic contaminants in terrestrial ecosystems with a variation of the bioavailable Pb fraction mainly due to soil characteristics, such as pH and organic substance concentration [13].

As regard the matrices of interest for the present study, the lead content in soil and wine can be influenced by different sources. The amount of lead assimilated by the grapevine and consequently present in the grapes is limited, suggesting a low natural content of this element [14,15]. However, it is worthwhile to remember that other lead sources must be considered, such as contributions due to human activity, contaminated materials used for the production, transport and storage of wine, and fallout of the metal present in the environment induced by precipitation [11]. In the present study the use of Pb isotope systematic, as geographical tracer for the soil-wine system, was investigated. A solid phase extraction procedure, by means of a chromatographic procedure, was used to separate Pb from the matrix prior to isotopes analyses performed with a multi collector inductively coupled plasma mass spectrometer, MC-ICP/MS. Moreover, external and internal mass dependent fractionation correction methods were tested to obtain reliable Pb isotopes ratios values. The developed analytical procedure was applied to soil and wine sample matrices. In particular, soils coming from three vineyards of the Modena province, one located in the south hill area and two in the north in-plain one, were subject to sampling procedure for two years to test the suitability of the isotopic marker as tracer. In addition, wine samples, obtained from the local Lambrusco wine Consortium and representative of the PDO production area, were tested according to the developed analytical procedure for the isotopic ratio determination.

## 2. Materials and Methods

### 2.1. Reagents and Standards

High purity deionized water, type I, was obtained by means of a Milli Q Element system (Millipore, Bedford, MD, USA). Physical and chemical parameters for Type I water comply with ASTM and ISO 3696 specification.

Suprapur 37% HCl (Merck, Darmstadt, Germany) and ultrapure 65% HNO_3_, the latter obtained from analytical grade HNO_3_ (CarloErba, Milan, Italy) by means of a DST 1000 sub-boiling system (Savillex, Eden Praire, MN, USA), were used for sample preparation and washing procedure.

Standard reference materials, NIST SRM 981 and NIST SRM 997, were purchased from National Institute of Standards and Technology (NIST). The certified values of the NIST SRM 981, are as follows: ^204^Pb/^206^Pb = 0.059042 ± 0.000037, ^207^Pb/^206^Pb = 0.91464 ± 0.00033, ^208^Pb/^206^Pb = 2.1681 ± 0.0008 (uncertainties are expressed as twice standard deviations: u = 2s). The certified value of the NIST SRM 997, ^203^Tl/^205^Tl, is 2.38714 ± 0.00101. Both standards were solubilized in 65% HNO_3_, and then diluted with 3M HCl to a final concentration of 200 μg kg^−1^ for Pb and 100 μg kg^−1^ for Tl, respectively. Single element Pb standard solution, 1000 ± 2 mg kg^−1^, used to determine total Pb concentration, was obtained from Merck (Darmstadt, Germany).

The Eichrom SR-B100-S (50-100um) resin, used for Pb separation, was purchased from Eichrom Laboratories (Bruz, France). The preparation procedure of the resin suspension is detailed in a previous work [16]. All the standard and sample solutions were gravimetrically prepared by using a Mettler AE200 analytical balance (Mettler Toledo AG, Greifensee, Switzerland) with ± 0.0001 g sensitivity.

### 2.2. Soil and Wine Samples

Soil samples, examined in this study, relate to three farms, A, B, and C located in Carpi, Bomporto and Savignano, situated in the Modena (Italy) province, respectively. The province of Modena is located in middle of the Emilia Romagna region in the north of Italy. The land characteristics vary from an in-plain area, located in the north-center of the province, to moderate/medium hill, in the southern part of the territory close to Appennini mountains. Furthermore, the northern, left and right boundaries are closed from the Po, Secchia and Panaro rivers, whose alluvial basins have a deep influence on the pedo and lithological characteristics of the territory. The sampling procedure and preliminary treatments are described in a previous work [17]. Briefly, in different sampling periods, September 2009 (second sampling campaign) and April 2010 (third sampling campaign), a representative number of soil samples were collected. Each soil sample was collected till a deep of 60 cm by using a single gauge auger set. Successively, after discarding the first 10 cm, the core was divided into five aliquots and labeled as follow: *a* from 10 to 20 cm, *b* from 20 to 30 cm, *c* from 30 to 40 cm, *d* from 40 to 50 cm and *e* from 50 to 60 cm.

For the present study, 36 soil samples, belonging to the second and third sampling campaigns, were subjected to Pb isotope ratio analyses. Moreover, relatively to farms A and B, located on the plain, where soil is more homogeneous, only the middle soil fraction *c* was taken into consideration. On the contrary, relatively to farm C, located in the hilly area, the highest *a* and the lowest *e* fractions were considered.

Finally, a soil sample was randomly selected as control soil and used to optimize the analytical method and to verify the repeatability and the reproducibility of the entire analytical procedure.

As far as the wine samples are concerned, the Lambrusco Consortium of Modena provided us 55 bottles of Italian wines. In particular, the following varietals were considered: Lambrusco Grasparossa di Castelvetro PDO (18 samples), Lambrusco di Sorbara PDO (14 samples), Lambrusco Salamino Santa Croce PDO (6 samples), produced in the province of Modena, and in addition, Lambrusco Mantovano (10 samples), from the province of Mantova, and Lambrusco Reggiano (7 samples), from the province of Reggio Emilia.

### 2.3. Sample Pre-treatment

A microwave acid digestion system, Ultrawave (Milestone, Bergamo, Italy), equipped with a five-position rack for 40 mL quartz vessels, was used to leach out Pb from the soil, while digestion of wine samples was carried out by cold mineralization [5]. The autoclave apparatus consists of a single reaction chamber where samples are placed simultaneously for microwave digestion under high temperature and pressure conditions. Sample mineralization was carried out on batch of five samples and on a total sample aliquot of roughly 5 g. In the case of soil samples, an aliquot of 1 g of soil and 10 mL of 10% HNO_3_ (*v*/*v*) were subjected to the microwave heating program reported in Table S1 (reported as Supplemental Material). The power of the instrument magnetron, 1500 W, is automatically managed by the Ultrawave, to obtain a heating ramp in accordance with the theoretical trend based on the set parameters as reported in Table S1. At the end of the microwave cycle, samples were filtered, through a 1.20 μm and 0.45 μm pore size filters, to obtain a clear solution.

Concerning wine samples, 15 mL of wine and 5 mL of 65% HNO_3_ were left to react, inside PFA tube, for at least 12 h. During this time, the temperature of the solution increased due to acid-ethanol reaction. A clear final solution was obtained without any visible residue.

### 2.4. Pb Concentration Measurement

Total Pb concentration, in all sample solutions, was measured by quadrupole-based inductively coupled plasma mass spectrometer, ICP/QMS (XSeries II model, Thermo Fisher Scientific, Bremen, Germany). Instrumental parameters and lead concentrations in soil and wine samples are reported in Tables S2, S3, and S4, respectively (*reported as* *Supplemental Material*).

### 2.5. Pb Separation by SPE

Owing to the presence of high levels of total dissolved solids (TDS) in the extracted soil samples and to the necessity of having a sample matrix as close as possible to the NIST standard, lead was separated from the matrix. As a consequence, a solid phase extraction (SPE) procedure was applied and optimized for the maximum Pb recovery. A series of recovery tests, with the NIST SRM 981 standard solution and with a solution obtained from the control soil sample, were carried out. In addition to Pb, the Tl content was also measured in the SPE eluted solution.

For the Pb NIST SRM 981 standard, a 400 μg kg^−1^ solution was prepared and subjected to separation; an average concentration of the SPE eluted solution of 376 ± 25 μg kg^−1^ was obtained and the recovery of the extraction process was 94 ± 8%. Moreover, Pb isotopes determination showed that no fractionation phenomena occurred during the separation.

In order to perform matrix removal from the samples in the same condition as for the standard, i.e., same initial Pb concentration, samples were diluted on the basis of their total Pb content. Repeated Pb separations of the soil control sample and successive ICP/QMS determinations confirmed that SPE procedure allowed to completely remove Tl, being the measured values less than the ICP/QMS detection limit (DL = 0.001 μg kg^−1^) for this element.

Lead separation was carried out with an in-house made solid phase extraction column using 1.5 mL capacity “Extract Clean Reservoir” (Alltech, Milan, Italy) loaded with 0.8 mL of Sr/Pb Eichrom resin suspension. The resin suspension was prepared as follow: 10 g of resin were conditioned in a 100 mL PFA bottle with 50 mL of HNO_3_ 1% *w*/*w*. After overnight soaking, the liquid phase was removed and the bottle was refilled with fresh HNO_3_ 1% *w*/*w* until the final volume of the solution was close to 100 mL. The suspension was stored at room temperature. Before each analysis, the bottle was shaken with a variable speed rotator (Rotator SB3, Stuart, Staffordshire, UK) for at least 30 min set at 10 rpm.

The content of Pb in the loaded soil sample was in the 4 to 5 μg range, while, for wine sample, ranged from 0.2 to 1 μg. Since the retention of Pb by the resin is very high in nitric acid, with a concentration ranging from 0.1 to 10 M, 6 M HCl was used as stripping solvent [18,19]. Table 1 reports the scheme of the separation procedure. The resulting solution was diluted with water, to reduce HCl concentration to 3 M, along with a Tl standard solution (NIST SRM 997) to obtain a 100 μg kg^−1^ and 50 μg kg^−1^ final Tl concentration for soil and wine samples, respectively. All the solutions were collected in PFA tubes and analyzed within few days.

### 2.6. Pb Isotope Ratios Determination

Lead isotopic ratios measurements were carried out by a multi collector inductively coupled plasma mass spectrometer (MC-ICP/MS) Neptune (Thermo Fisher Scientific, Bremen, Germany). This double focusing, forward Nier-Johnson type, mass spectrometer, consisting of an electrostatic sector and an orthogonal magnetic sector, is equipped with eight movable Faraday collectors and a fixed central one. Data acquisition was performed in low resolution on a flat top peak signal for all the recorded ions and, regarding to the Faraday cups, in static mode.

The instrumental set up parameters are reported in Table S5 (*reported as* *Supplemental Material*). The setting of the instrument, namely tune procedure, was daily performed to maximize instrumental stability and the shape of the flat top peak. Two different sample introduction systems were used on the basis of sample Pb concentration. In particular, for wine samples, where Pb concentrations is lower than 100 μg kg^−1^, an APEX IR desolvating apparatus (Elemental Scientific Instruments, Huntingdon, UK) was used; while a combined cyclonic and Schott type spray chamber was used for more concentrated extracted soil solutions.

A [blank_1_/standard_1_/blank_2_/sample_1_/blank_3_/standard_2_/blank_4_] bracketing sequence was always adopted to check and correct for any instrumental drift. In the present study, the measured ^208^Pb/^206^Pb and ^207^Pb/^206^Pb ratios (^20x^Pb/^20y^Pb ratios) were corrected for the instrumental mass bias by different mathematical approaches as reported in the next section. In addition, to correct for the isobaric interference of ^204^Hg, that overlaps with ^204^Pb, the ^202^Hg was monitored [20]. The MC-ICP/MS instrumental condition, in particular the plasma argon gas, showed a very low contribution of the mercury and therefore the ^204^Hg/^204^Pb ratio resulted to be negligible.

### 2.7. Mass Bias Correction Methods

When dealing with IRs determinations, the measured raw data must be properly corrected for instrumental biases, including the mass dependent fractionation phenomena occurring in the plasma interface and consisting in a different transmission of the isotopes of the same element towards the spectrometer. In particular, heavier ions are more transmitted than lighter ones [21,22].

In order to verify the suitability and effectiveness of the analytical procedure on the accuracy of the final data, evaluated with respect to international reference standards, different correction methods were applied. The approaches used to correct the measured intensities of each collected ions for mass discrimination might involve an "external" or "internal" type of correction [23]. In both cases, it is possible to use an isotopic pair of a different element for the correction of the mass bias. In the specific case of lead, it is possible to use the ^205^Tl/^203^Tl isotopic ratio since the mass fractionation is mostly independent from the chemical properties of the element and mainly related to the mass values. However, to use an isotopic pair of a different element from the one of interest, it is necessary to establish whether to consider the isotopes fractionation coefficient, *f*, for the two elements, identical or different [24]. Moreover, in addition to the use of a different element reference system, the mass dependent fractionation bias can also be evaluated by applying an external correction procedure through a couple of lead isotopes, ^20x^Pb/^20y^Pb, measured on a reference standard solution and using the so-called C_factor_ method. The procedures for the instrumental mass bias correction, namely external correction with a pair of Pb isotopes (method *a*), internal correction with ^205^Tl/^203^Tl considering *f*_Pb_ = *f*_Tl_ (method *b*) and the internal correction with ^205^Tl/^203^Tl but with different fractionation factor, *f*_Pb_ ≠ *f*_Tl_, (method *c*) are presented and briefly discussed. Although, the mathematical procedures are more or less well established, for the instrumental mass bias correction of MC-ICP/MS data, their efficiency is often instrument dependent and therefore it is useful to consider pros and cons of each analytical approach. The methods, as well as the mathematical development of the correction procedures, are reported as separate contribute (Appendix A), while in the discussion section the main results are analyzed.

### 2.8. Statistical Analysis

Data comparison was performed using a Student t-test. An alpha level of confidence of 0.05 was used for all statistical tests performed by the “data analysis” macro of Excel^®^ 2011 for Mac, version 14.0.0, Microsoft Corp. Redmond, WA, U.S.A.

## 3. Results and Discussion

### 3.1. Instrumental Mass Dependent Fractionation Correction

Despite all Pb isotopes were measured by MC-ICP/MS, the ^204^Pb isotope, owing its low natural abundance, produced data that were not reliable as those measured for the other ones. As a consequence, results and discussion refer to the ^208^Pb/^206^Pb and ^207^Pb/^206^Pb isotope ratios.

The choice of the proper instrumental mass bias correction method represents a key point for the development of the analytical procedure. In particular, method (*a*) was directly applied for the correction of the analyzed samples, while methods (*b*) and (*c*) were at first tested on NIST SRM 981 standard solutions and successively the extrapolated regression coefficients used to correct the samples. Specifically, method (*a*) did not provide results as precise as the remaining two methods.

In particular, precision of the data was evaluated starting from the control sample values corrected with methods (*a*), (*b*) and (*c*), respectively (Table S6, reported as Supplemental Material). The t-Student’s test was applied to the averaged values of ^208^Pb/^206^Pb ratio. For methods (*a*) and (*b*), the data resulted to be statistically different (t_calc_ = 4.82, t_tab_ (df = 34, α = 0.05) = 2.04) as well as for method (*a*) and (*c*) (t_calc_ = 3.66, t_tab_ (df = 34, α = 0.05) = 2.04). Concerning the comparison among the ^207^Pb/^206^Pb ratios, any statistically significant difference was detected. Based on these results, and in addition to the fact that an internal correction method is preferred towards an external one (see Appendix A), method (*a*) was not considered as a suitable approach for the mass bias correction.

On the contrary, the two internal correction approaches, considering the fractionation coefficients of Pb and Tl equal (*b*) and different (*c*), allow obtaining the results reported in Table 2.

**Figure 1:**
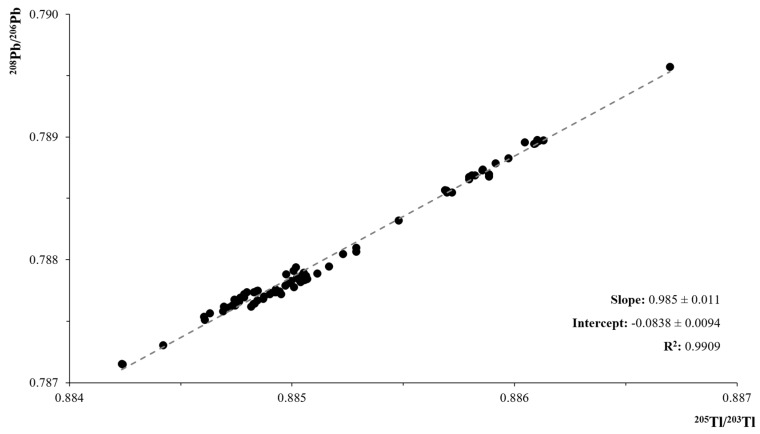
Plot of ln (208Pb/206Pb) vs. ln (205Tl/203Tl) data for the NIST SRM 981and NIST SRM 997 standards, measured in the July–September 2016, January–February 2017 and May 2019 sessions.

In all the experiments, the precision of the isotopic ratios resulted to be better than that of the certified NIST SRM 981 value, as confirmed by the uncertainties reported in the same table. In addition, it can be observed that, with the correction method (***c***), the calculated uncertainty of the data is even better for ^207^Pb/^206^Pb ratio and the value remains statistically equivalent to the NIST data as stated by the t-test result, t_calc_ = 0.14 t_tab_(df = 23, α =0.05) = 2.07. The same considerations cannot be done for the ^208^Pb/^206^Pb ratio since the Student’s t-test resulted to be negative, t_calc_ = 17.67 t_tab_(df = 24, α = 0.05) = 2.06. On the other hand, data of Table 2, obtained from the ln ^208^Pb/^206^Pb vs. ln ^205^Tl/^203^Tl plot, Figure 1, are in perfect accordance with literature values [24], some of which are listed in Table 3.

Therefore, owing to the smaller uncertainties obtained with method (***c***), this mathematical approach was applied for the correction of the mass bias in the measured soil and wine samples.

In order to monitor the performance of the method over time, a control sample was used. In particular, to check for the reproducibility of the measurement steps, the control sample was subjected to the entire analytical procedure for each batch of microwave treatment. The control sample solutions were subjected to the five MC-ICP/MS working sequences and at least one solution was measured in two consecutive sessions as shown in Figure S1 (reported as Supplemental Material).

The values relating to the control soil sample, both as absolute mean value (^208^Pb/^206^Pb = 2.07474 ± 0.00029 and ^207^Pb/^206^Pb = 0.83992 ± 0.00016, u = 2s, *n* = 18) and as temporal distribution for the ^207^Pb/^206^Pb isotope ratio, constitute an objective reference of the relative trustworthiness of the measured data. The experimental values, obtained from measurements carried out in different sessions, resulted to be in good agreement, in fact the difference between each couple of values is of the same order of magnitude as the measurement uncertainty. This demonstrates how the internal correction method with Tl, considering different fractionation coefficients, manages to compensate the long-term instrumental instabilities that can occur when the measuring sessions insist over a long-time frame.

### 3.2. Pb Concentration in Soil and Wine Samples

**Figure 2:**
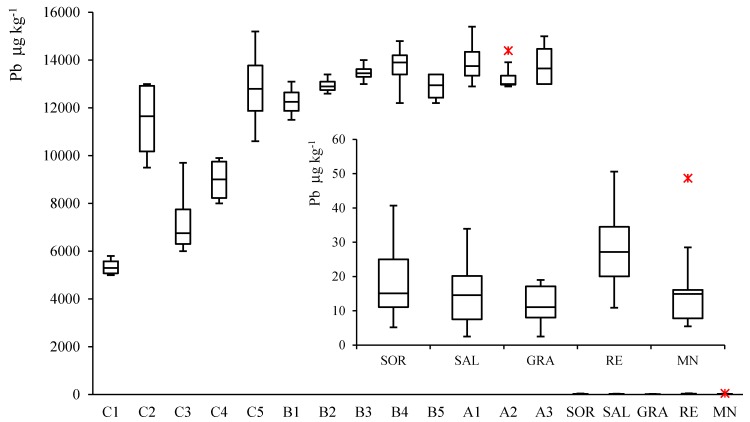
Box and whiskers plot representation of the lead concentrations data for soil, C1—C5, B1–B5, A1—A3 and wine samples, Lambrusco Sorbara, SOR; Lambrusco Salamio, SAL; Lambrusco Grasparossa, GRA; Lambrusco Reggio Emilia, RE; Lambrusco Mantova, MN, respectively.

The box and whiskers plot, reported in Figure 2, shows the lead concentrations of the soil and wine samples investigated in this study. Soil data, reported in Table S3, were grouped according to the different depth and sampling period, for site C, and repeated sample of different sampling period, for sites A and B. Wine data, reported in Table S4, are summarized on the base of the different Lambrusco varietal or production zones.

The plot shows that the lead content, detected in the different soil samples, is significantly different from that measured in wines. In fact, the difference between the two matrices is approximately three order of magnitude, i.e., mg kg^−1^ for the soils (11.7 ± 2.8 mg kg^−1^, mean ± SD, *n* = 54) and g kg^−1^ for wines (16.8 ± 11 g kg^−1^, mean ± SD, *n* = 55), as better depicted in the enlarged view of Figure 2.

In particular, the lead content determined in the soil samples taken in the hilly area, C1–C5, highlights a considerable intra-site variability while the values are more or less unchanged between the two sampling campaigns (see data in Table S3). On the contrary, the lead concentration measured in samples from the plain areas, B1–B5 and A1–A3 (see data in Table S3) is, on average, higher with respect to the hill ones, and almost constant both for intra-site and geographical areas.

Although soil represents nowadays the main Pb depositary, the measured data range in the 5 ÷ 18 mg kg^−1^ interval. These values are usually encountered in areas remote from human activities and are similar to concentrations found in rocks with an average range comprised from 5 to 25 mg kg^−1^, in agreement with the not polluted cultivated soil content that for lead ranges from the 10 to 100 mg kg^−1^ interval [33].

Regardless the Pb concentration in wines (see data in Table S4), all investigated samples were in conformity with the health safety standards, being the permissible concentration of Pb in oenological products limited by 0.15 mg L^−1^ (this value is close to 0.149 mg kg^−1^). This level was established in 2006 by the OIV (International Organization of Vine and Wine) as new maximum limit and is still in effect today [34].

### 3.3. Determination of Isotopic Ratios: Soils and Wines

**Figure 3:**
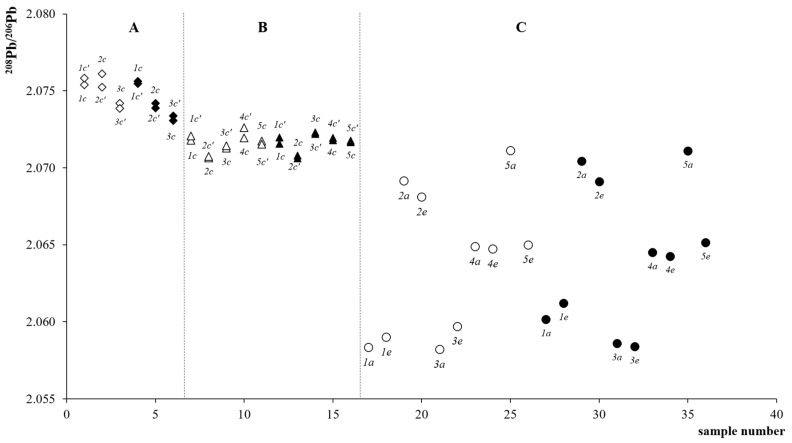
Plot of the ^208^Pb/^206^Pb isotopic ratio data for the soil samples obtained with the internal correction considering fPb ≠ fTl. Sample label indicates position inside vineyard or depth: Increasing depth is from *a* to *e*. A, in plain area Carpi, (◇) second sampling, (◆) third sampling; B, in plain area Bomporto, (△) second sampling, (▲) third sampling; C, hill area Savignano, (◯) second sampling, (●) third sampling. Sizes of the symbols are equivalent to the uncertainty interval associated to the plotted data.

Figure 3 shows the values of the isotopic ratio ^208^Pb/^206^Pb for soil samples coming from vineyards, A and B, located on the plain and C, located in the hill and sampled during the second, September 2009, and third, April 2010, campaigns of sampling [17].

With regard to data measured for the hill site C, the ^208^Pb/^206^Pb ratio ranges from 2.07112 to 2.05821, for samples collected in September 2009, and between 2.07110 and 2.05839 for those collected in April 2010. The two distributions are almost similar and, in terms of isotopic composition, both show intra-site variability. Nevertheless, taking into account the uncertainty associated to the control sample isotopic data, equal to 2.9·10^−4^ for the ^208^Pb/^206^Pb ratio, the two set of data result not statistically different, p(|t| ≥ 0.31 = 0.76). Concerning the sampling positions reported in Figure 3, it is possible to observe that sites *1* and *3* are in some way separated from locations *2*, *4* and *5*.

This intra-site separation is comparable to that previously highlighted by considering the ^87^Sr/^86^Sr ratio [16] and confirms the discriminating potentialities of the lead isotope signature in differentiating soils on the basis of their origin and/or composition. In particular, locations *1* and *3* have the lowest ^208^Pb/^206^Pb ratio; location *4* has intermediate values while locations *2* and *5* have the highest isotopic ratio. In addition, sampling position *5* shows a substantial variation of the IRs between the *a* and *e* depths, indicating a difference of composition alongside the sampling depth and confirmed by the data of the third sampling. Moreover, data reported in Figure 3 for site C show, on average, a decreasing trend of the IRs with the increasing of the sample depth. Similar results were obtained from the analysis of the ^207^Pb/^206^Pb data.

As regard the vineyards located on the plain, a restricted intra-field variation of the IRs is observed. In fact, in the case of site B the ratio ^208^Pb/^206^Pb ranges from 2.07065 to 2.07206 and from 2.07062 to 2.07231 for September 2009 and April 2010, respectively; while the vineyard located in site A is characterized by a ^208^Pb/^206^Pb ratio ranging from 2.07385 to 2.07612 and from 2.07307 to 2.07561 for September 2009 and April 2010, respectively. In the case of farm B, data are mostly constant within the investigated area. Lead isotope ratios for site A are almost identical to those measured for site B, with the exception of the *1c* sampling point that is characterized by a higher value. The greater uniformity of the data for plain area is not surprising, since also the values of ^87^Sr/^86^Sr produced almost the same response although the reasons for this behavior might be different [16].

In general, for soil samples, the obtained values of the lead content and the isotopic ratio, are coherent with the literature data of other European agricultural lands [14,35] and for farmed soils in northern Italy [36].

**Figure 4:**
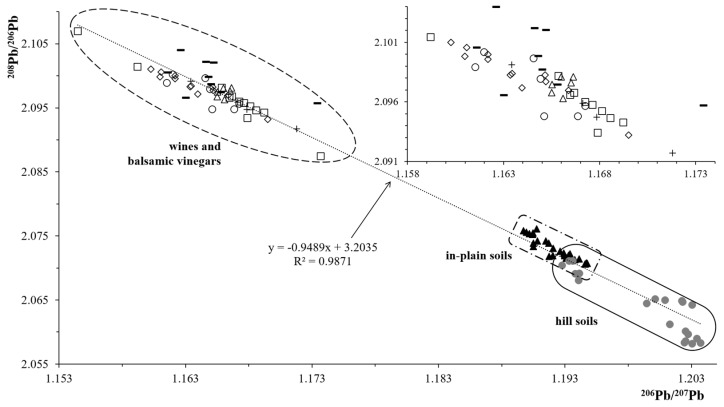
Plot of the ^206^Pb/^207^Pb *vs.* ^208^Pb/^206^Pb data for soil and wine samples. (●) Hill soils C, (▲) in-plain soils A and B, (◇) Lambrusco di Sorbara, SOR; (☐) Lambrusco Grasparossa, GRA; (+) Lambrusco Salamino, SAL; (◯) Lambrusco Reggio Emilia, RE; (△) Lambrusco Mantova, MN; and (**−**) italian balsamic vinegars [36].

Figure 4 shows the values of ^208^Pb/^206^Pb vs. ^206^Pb/^207^Pb of soil and wine samples analyzed in this work in addition to literature data from Italian balsamic vinegars [37]. With this representation, three isotopes plot, it is possible to highlight different behaviors on the basis of the data distribution. In particular, the IRs form a cluster when there is only one source of the element, while they tend to linearly spread when there are more lead sources entering the system [11].

The trend of the data outlined by Figure 4, immediately shows a peculiar situation where the soil values are grouped and mainly located in the right lower part of the plot, showing a clustered distribution for hill samples at high ^206^Pb/^207^Pb and low ^208^Pb/^206^Pb values typical of uncontaminated soils [11]. On the contrary, in plain soils and wines data are linearly distributed on the three isotopes plot at higher ^208^Pb/^206^Pb values confirming the presence of different lead sources. The main evidence, coming from the data of Figue 4, is the absence of any correlation between wines and soils in terms of geographical origin. In particular, wine samples are positioned at values of IRs ranging from 2.08751 to 2.10697 for the ^208^Pb/^206^Pb ratio and result higher with respect to the soil ones that are comprised in the 2.05821 to 2.07612 interval.

Wine samples IRs well agree with literature data concerning different oenological products such as the Italian balsamic vinegars [37].

The absence of a geographic link between soil and wine confirms that, contrary to the uptake mechanism of Sr by vine roots, the vine plant slightly or probably not absorbs lead. Although some authors admit the possibility for the grapevine to assimilate lead [38], other researchers have recently concluded that this possibility is minimal or negligible, and the presence of Pb in wines is a direct consequence of the universal presence of the element in the environment [39]. Our results confirm the characteristics of lead as environmental monitoring marker, rather than geographical traceability indicator.

Therefore, both for soil and wine samples, the end members for the lead presence are more than one; some of them prevail for the wines while others for the soils leading to different values of the IRs as shown in Figure 4. As regards the presence of lead in wine, it is possible to hypothesize a contribute from the production processes in addition to the Pb fraction coming from the waste combustion with deposition of ashes dispersed in the atmospheric aerosol [40].

As far as the possibility of differentiating wine samples of different geographical origin is concerned, on the basis of this data set, it is not possible to separate any of the measured vine varietals. In fact, as shown in Figure 4, Lambrusco Mantova samples are well grouped but are embedded within Lambrusco Grasparossa and Lambrusco Reggio Emilia wines.

Therefore, the trend highlighted in the three isotopes plot confirms the presence of several lead sources with respect to soils data but at the moment the different end members are still unknown and more work must be done to characterize the environmental lead signature. Indeed, it can be assumed that the main source of lead in wine is attributable to an anthropic origin, probably ascribable to the atmospheric fallout of the suspended particulate, rather than deriving from the soil or from the contact with metals during the production processes in the cellar. Experimental data do not show peculiar trends or distributions of the IRs neither according to the Lambrusco wine varietals nor with respect to their geographical origin.

In summary, lead’s isotope ratio measurements, carried out with MC-ICP/MS, are affected by mass dependent fractionation phenomena. The mathematical method that better corrects for mass bias resulted to be the internal correction method based on the use of Tl isotopes, considering different the isotopes fractionation coefficient, *f*, for Pb and Tl.

Concerning the potentialities of Pb isotope ratio as geographical tracer, with particular reference to Italian Lambrusco PDO wine, results show that anthropogenic sources of lead probably interfere in the soil-wine system and in the varietal differentiation.

## Figures and Tables

**Table 1:** Separation scheme for Pb extraction.

Step	Eluent	Volume (mL)	Function
1	H_2_O	2	Resin pre-washing
2	8M HNO_3_	12	Resin conditioning
3	Sample loading	1.5–4.5 (soil) 20 (wine)	Sample loading
4	8M HNO_3_	4	Resin rinsing
5	H_2_O	13	Resin rinsing (Sr elution)
6	6M HCl	10 (soil) 3 (wine)	Pb elution

**Table 2:** Lead I.R. values obtained with different correction methods for the NIST SRM 981 standard solutions and respective uncertainties, u = 2s.

NIST SRM 981 ^#^			
	I.R.	^208^Pb/^206^Pb	^207^Pb/^206^Pb
Corr. Method			
*Certified values*		2.1681 ± 0.0008	0.91464 ± 0.00033
(*b*) *f_Pb_ = f_Tl_*		2.16665 ± 0.00023	0.914624 ± 0.000057
(*c*) *f_Pb_ ≠ f_Tl_*		2.16664 ± 0.00021	0.914645 ± 0.000021

^#^ 97 measures, collected in July–September 2016, January–February 2017 and May 2019 sessions, are averaged for method (**b**) while 84 data were used for method (**c**).

**Table 3:** Lead isotopic ratios values reported for NIST SRM 981 measured by TIMS and/or MC-ICP/MS.

Authors	Mass Spectrometer ^#^	^208^Pb/^206^Pb	^207^Pb/^206^Pb	^206^Pb/^204^Pb	^207^Pb/^204^Pb	^208^Pb/^204^Pb
Todt et al. [25]	TIMS	2.16701 (43)	0.91459 (13)	16.9356 (23)	15.4891 (30)	36.7006 (112)
Galer [26]	TIMS	2.16771 (10)	0.914750 (35)	16.9405 (15)	15.4963 (16)	36.7219 (44)
Thirlwall [27]	TIMS	2.16770 (21)	0.91469 (7)	16.9409 (22)	15.4956 (26)	36.7228 (80)
Hirata [28]	MC-ICP/MS—*Plasma 54*	2.16636 (82)	0.914623 (37)	16.9311 (90)	15.4856	36.6800 (210)
Rehkaemper, Halliday [29]	MC-ICP/MS—*Plasma 54*	2.16677 (14)	0.91469 (5)	16.9364 (55)	15.4912 (51)	36.7219 (44)
White et al. [24]	MC-ICP/MS—*Plasma 54*	2.1646 (8)	0.91404	16.9467 (76)	15.4899 (39)	36.6825 (78)
Rehkaemper and Mezger [30]	MC-ICP/MS—*IsoProbe*	2.16691 (29)	0.91459 (13)	16.9366 (29)	15.4900 (17)	36.7000 (23)
Reuer et al. [31]	MC-ICP/MS—*IsoProbe*	2.16639 (304)	0.91460 (18)			
Weiss et al. [19]	MC-ICP/MS—*IsoProbe*	2.16767 (63)	0.914767 (120)	16.9413 (39)	15.4974 (51)	36.7239 (115)
Gallon [32]	MC-ICP/MS—Neptune Spray chamber	2.16606 (46)	0.91441 (28)	16.9315 (118)	15.4824 (91)	36.6746 (220)
Gallon [32]	MC-ICP/MS—Neptune Apex	2.16607 (30)	0.91451 (20)	16.9308 (54)	15.4835 (24)	36.6733 (79)
**This study**	MC-ICP/MS—Neptune Spray chamber and Apex	2.16664 (21)	0.914645 (21)	16.9310 (32)	15.4858 (30)	36.6834 (70)

^#^ The TIMS data are based on double and triple spike measurements. Uncertainties shown in parenthesis refer to the least significant digits and are all given as u = 2 s.

## Supplementary Materials

Supplementary Materials can be found online. Figure S1: ^207^Pb/^206^Pb isotopic ratio data for the separated control soil samples (N = 18) obtained with the internal correction considering f_Pb_ ≠ f_Tl_. (◆) data of 14/06/2016, (■) data of 04/07/2016, (▲) data of 13/09/2016, (●) data of 26/09/2016, (◆) data of 17/01/2017, (- -) mean value. Linked values correspond to the same control sample measured in different sessions; Table S1: Microwave heating program used to leach out Pb from soil samples; Table S2: Operating parameters implemented on the ICP-MSq, spectrometer for Pb content determination; Table S3: Pb concentration, mg kg^−1^, and isotope ratios determined in soil samples relatively to the investigated A, B and C zones. The concentration values are characterized by an RSD%**^#^** of 3%. Uncertainty**^#^** associated to ^208^Pb/^206^Pb and ^206^Pb/^207^Pb values are ± 0.00029 and ± 0.00016, respectively; Table S4: Pb concentration, μg kg^−1^, and isotope ratios determined in Lambrusco wine samples. Concentration values are characterized by an RSD%**^§^** of ±10%. Uncertainty**^§^** associated to ^208^Pb/^206^Pb and ^206^Pb/^207^Pb values are ± 0.00029 and ± 0.00016, respectively; Table S5: Operating parameters used to measure the isotopic ratio with the Neptune MC-ICP/MS spectrometer; Table S6: Averaged lead I.R. values (n = 18) obtained with different mass bias correction methods determined on the control soil sample and respective uncertainties, u = 2 s.

## Abbreviations

CRM	Certificate Reference Material
DL	Detection Limit
ICP/QMS	Inductively Coupled Plasma Quadrupole Mass Spectrometry
IR	Isotope Ratio
ISO	International Organization for Standardization
MC-ICP/MS	Multi Collector Inductively Coupled Plasma Mass Spectrometry
NIST	National Institute of Standard and Technology
OIV	International Organization of Vine and Wine
PDO	Protected Designation of Origin
PFA	Perfluoroalkoxy
PGI	Protected Geographical Identification
SPE	Solid Phase Extraction
SRM	Standard Reference Material
TDS	Total Dissolved Solids
TIMS	Thermal Ionization Mass Spectrometry
TSG	Traditional Specialty Guaranteed

## Appendix A. Methodological procedure for the instrumental mass bias correction in MC-ICP/MS isotope ratio determinations

### Appendix A.1. Mass Bias Correction with C_factor_ (Method a)

The fractionation factor is determined from the lead IRs measurements of the NIST CRM 981 standard solutions. In particular, the C*_factor_* is calculated by the ratio between the certified value, “*std true*”, and the measured one, “*std meas*”, as reported by Equation (A1).

(A1) $$C_{factor}=I.R._{std\;true}/I.R._{std\;meas}$$

The C*_factor_* is then applied to the unknown sample in order to calculate the Pb isotopic ratio of the sample, Equation (A2).

(A2) $$CI.R._{smpl\;corr}=I.R._{smpl\;meas}\;\cdot \;C_{factor}$$

Although the method is quite simple and easily applicable as routine procedure, standards and samples must be measured at different times and two main points must be considered. The former is the necessity of a matrix matching between standards and samples solutions and the latter is the constancy of the instrument setup during the measuring cycle. While the matrix matching represents an experimental condition relatively simple to obtain, the constancy of the instrument performances is somewhat more complicate to realize and special measuring sequences, such as the bracketing approach, must be implemented to overcome the time dependent instrumental drift.

### Appendix A.2. Mass Bias Internal Correction with ^205^Tl/^203^Tl Isotope Ratio (Methods b and c)

The instrumental mass bias was corrected by means of an exponential law to obtain IRs characterized by great precision and accuracy, as reported by other authors [19,24,32,41].

The starting general formula, applied to a generic couple of Pb isotopes is:

(A3) (P20xbP20yb)true=(P20xbP20yb)meas[M20xM20y]fPb

where the subscript "true" indicates the true value or the certified isotope ratio, while “meas” indicates the measured one; *M* is the atomic mass of the isotopes and f_Pb_ is the fractionation coefficient for the considered isotopes [41]. The possibility to correct for the instrumental mass bias by internal correction overcomes, on one hand, the limits of the C_factor_ procedure but, on the other hand, introduces some more conceptual and mathematical complications.

In fact, by applying Equation (A3) to Pb and Tl isotopic ratios, the following relation is obtained:

(A4) ln(P20xbP20yb)meas=[ln(P20xbP20yb)true−fPbfTlln(M20xM20y)ln(M205M203)ln(T205lT203l)true]+fPbfTlln(M20xM20y)ln(M205M203)ln(T205lT203l)meas

Equation (A4), that can be written for both the ^207^Pb/^206^Pb and ^208^Pb/^206^Pb ratios, can be further rearranged on the basis of two different assumptions, namely *f_Pb_* = *f_Tl_* or *f_Pb_* ≠ *f_Tl_*, respectively.

If the former assumption is considered, *f_Pb_* = *f_Tl_*, then the final equation for the ^20x^Pb/^20y^Pb isotopic ratio determination can be written as follows:

(A5) (P20xbP20yb)true=(P20xbP20yb)meas[M20xM20y]ln[(T205lT203l)true(T205lT203l)meas]ln(M205M203)

The use of Equation (A5) to calculate the corrected isotopic ratio, for the real sample, has the advantage that, with respect to the external correction method, all the isotopes in standards and samples are measured at the same time. However, the assumption that the fractionation factors are equal might produce some artifacts on the final results [22]. As a consequence, the second assumption, *f_Pb_* ≠ *f_Tl_*, must be considered and the experimental measurements planned with a bracketing sequence (blank-std-blank-sample)_n_. Approximating Equation (A4) to a linear relation and plotting the logarithmic terms of the resulting Equation (A6), the slope s, Equation (A7), and the intercept q, Equation (A8), can be calculated.

(A6) ln(P20xbP20yb)meas=q+s ln(T205lT203l)meas

(A7) $$slope=s=\frac{f_{Pb}}{f_{Tl}}\frac{ln\left(\frac{M_{20x}}{M_{20y}}\right)}{ln\left(\frac{M_{205}}{M_{203}}\right)}$$

(A8) intercept=q=[ln(P20xbP20yb)true]−fPbfTlln(M20xM20y)ln(M205M203)ln(T205lT203l)true

Despite more accurate on theoretical basis, this mass bias correction approach has some drawbacks in its practical application. In fact, the *f_Pb_*/*f_Tl_* ratio must be firstly determined from the standard measurements and then applied to the sample ones [23,42,43]. Hence, also in this case the matrix matching, between standard and sample solutions, is a stringent pre-requisite to obtain reliable results.

Although the use of this method, to determine every isotope ratio, would be preferable thanks to its total independence from assumptions on mass bias factors, it remains widely assumed that the fractionation factor remains the same for all the isotope ratios of a given element. Therefore, once the corrected (^208^Pb/^206^Pb)*_true_* is obtained, from the log-linear regression between ln (^208^Pb/^206^Pb)*_meas_* and ln (^205^Tl/^203^Tl)*_meas_*, all the other Pb isotope ratios of interest can be determined by using the (^208^Pb/^206^Pb)*_true_* as internal correction term. 2.1. Subsection
