# Supplementary material for: Use of Lead Isotopic Ratios as Geographical Tracer for Lambrusco PDO Wines
Source: Molecules. 2020 Apr 2;25(7):1641. doi: 10.3390/molecules25071641 (PMC7180896; doi:10.3390/molecules25071641)
Supplement: Supplementary file 1 [file molecules-25-01641-s001.pdf]

Supplementary Material

Use of Lead Isotopic Ratios as Geographical Tracer for Lambrusco PDO Wines

Lisa Lancellotti, Simona Sighinolfi, Andrea Marchetti * and Lorenzo Tassi

Department of Chemical and Geological Sciences, University of Modena and Reggio Emilia, 41125 Modena, Italy; lisa.lancellotti@unimore.it (L.L.), simona.sighinolfi@unimore.it (S.S.), lorenzo.tassi@unimore.it (L.T.)

* Correspondence: andrea.marchetti@unimore.it; Tel.: +39-05-9205-8637

Table S1. Microwave heating program used to leach out Pb from soil samples.

**Time** (min)	**Temperature** (°C)
5	100
20	120
25	120

Starting conditions: N_2 pressure 30 Bar, temperature 25 °C.

Table S2. Operating parameters implemented on the ICP-QMS, spectrometer for Pb content determination.

Parameter	Setup value
Delay	20 s
Extraction[1]	−176.0 V
L1[1]	−1170 V
L2[1]	−80 V
Focus[1]	−10.0 V
D1[1]	−57.6 V
D2[1]	−146 V
Pole Bias[2]	−18 V
Sample gas[3]	0.72 mL min^{-1}
Hexapole[2]	−20 V
Forward[4]	1400 W
L3[1]	−195.3 V
Horizontal[5]	65 mm
Vertical[5]	475 mm
DA[1]	−28.2 V
Cooling gas[6]	13 L min^{-1}
Auxiliary gas[7]	1.08 L min^{-1}

[1] Potentials applied to lenses to focus the ion beam, [2] Potentials applied to quadrupole mass analyzer (Pole Bias) and hexapolar collision cell (Hexapole Bias), [3] Ar flow for the sample uptake, [4] Power of Radio Frequency applied to plasma torch, [5] Position of the plasma torch with respect to sample cone, [6] Plasma cooling gas, [7] Ar flow which contributes to the plasma formation.

Table S3. Pb concentration, mg kg^{-1}, and isotope ratios determined in soil samples relatively to the investigated A, B and C zones. The concentration values are characterized by an RSD%# of 3%. Uncertainty# associated to $^{208}Pb/^{206}Pb$ and $^{206}Pb/^{207}Pb$ values are ± 0.00029 and ± 0.00016, respectively.

	Second sampling September 2009				Third sampling April 2010			
**Farm**	**Sampling sites**	**Pb mg kg^{-1}**	**$^{208}Pb/^{206}Pb$**	**$^{206}Pb/^{207}Pb$**	**Sampling sites**	**Pb mg kg^{-1}**	**$^{208}Pb/^{206}Pb$**	**$^{206}Pb/^{207}Pb$**
C	1a	5.1	2.05834	1.20371	1a	5.0	2.06015	1.20253
	1e	5.5	2.05900	1.20342	1e	5.8	2.06121	1.20128
	2a	13	2.06916	1.19411	2a	12.9	2.07044	1.19277
	2e	9.5	2.06811	1.19407	2e	10.4	2.06911	1.19381
	3a	7.1	2.05821	1.20304	3a	9.7	2.05859	1.20253
	3e	6.0	2.05971	1.20273	3e	6.4	2.05839	1.20243
	4a	9.9	2.06488	1.20222	4a	9.7	2.06450	1.19946
	4e	8.3	2.06473	1.20228	4e	8.0	2.06426	1.20304
	5a	12.3	2.07112	1.19360	5a	15.2	2.07110	1.19330
	5e	10.6	2.06500	1.20089	5e	13.3	2.06515	1.20012
B	1c	12.5	2.07178	1.19331	1c	13.1	2.07156	1.19311
	1c′	11.5	2.07206	1.19341	1c′	12.0	2.07198	1.19328
	2c	13.0	2.07065	1.19463	2c	12.8	2.07080	1.19471
	2c′	13.4	2.07073	1.19476	2c′	12.6	2.07062	1.19459
	3c	13.5	2.07127	1.19377	3c	14.0	2.07231	1.19290
	3c′	13.4	2.07142	1.19412	3c′	13.0	2.07219	1.19341
	4c	12.2	2.07193	1.19295	4c	14.8	2.07180	1.19175
	4c′	13.8	2.07260	1.19261	4c′	14.0	2.07195	1.19203
	5c	12.5	2.07175	1.19324	5c	13.4	2.07167	1.19349
	5c′	13.4	2.07154	1.19312	5c′	12.2	2.07176	1.19332
A	1c	15.4	2.07538	1.19018	1c	12.9	2.07561	1.18996
	1c′	14.0	2.07582	1.18972	1c′	13.5	2.07548	1.19047
	2c	14.4	2.07612	1.19076	2c	12.9	2.07419	1.19149
	2c′	13.0	2.07524	1.19043	2c′	13.0	2.07389	1.19172
	3c	14.3	2.07419	1.19083	3c	15.0	2.07307	1.19207
	3c′	13.0	2.07385	1.19051	3c′	13.0	2.07337	1.19049

#The RSD% and uncertainty values, associated to the reported data, were evaluated by repeated and replicated measurements performed on the soil control sample.

Table S4. Pb concentration, μg kg^{-1}, and isotope ratios determined in Lambrusco wine samples. Concentration values are characterized by an RSD%§ of ± 10%. Uncertainty§ associated to $^{208}Pb/^{206}Pb$ and $^{206}Pb/^{207}Pb$ values are ± 0.00029 and ± 0.00016, respectively.

Wine#	Pb μg kg^{-1}	$^{208}Pb/^{206}Pb$	$^{206}Pb/^{207}Pb$	Wine#	Pb μg kg^{-1}	$^{208}Pb/^{206}Pb$	$^{206}Pb/^{207}Pb$
SOR	10.6	2.09717	1.16396	GRA	12.5	2.09344	1.16788
SOR	40.7	2.10057	1.16107	GRA	11.0	2.09660	1.16645
SOR	15.1	2.09827	1.16335	GRA	11.0	2.09601	1.16723
SOR	11.1	2.09839	1.16345	GRA	17.5	2.10697	1.15448
SOR	13.5	2.10101	1.16026	GRA	18.7	2.09678	1.16666
SOR	30.9	2.09983	1.16099	GRA	14.9	2.08751	1.17366
SOR	14.5	2.09957	1.16218	GRA	11.0	2.09465	1.16856
SOR	15.5	2.09998	1.16218	GRA	17.9	2.09820	1.16585
SOR	19.9	2.09696	1.16637	GRA	19.0	2.09576	1.16761
SOR	28.2	2.09825	1.16515	GRA	17.0	2.09524	1.16811
SOR	25.0	2.09770	1.16519	GRA	11.2	2.09430	1.16922
SOR	48.7	2.09321	1.16951	GRA	16.1	2.10145	1.15919
SOR	5.2	ND	ND	GRA	2.5	ND	ND
SOR	10.1	ND	ND	GRA	9.0	ND	ND
SAL	11.5	2.09590	1.16712	GRA	4.1	ND	ND
SAL	17.7	2.09472	1.16785	GRA	2.9	ND	ND
SAL	21.0	2.09170	1.17179	GRA	5.1	ND	ND
SAL	33.9	2.09913	1.16342	GRA	5.5	ND	ND
SAL	6.2	ND	ND	MN	30.1	2.09677	1.16550
SAL	2.5	ND	ND	MN	15.4	2.09629	1.16609
RE	37.6	2.09567	1.16724	MN	14.9	2.09809	1.16600
RE	27.2	2.09892	1.16152	MN	16.2	2.09762	1.16652
RE	31.4	2.09481	1.16510	MN	13.7	2.09747	1.16552
RE	10.9	2.09482	1.16685	MN	15.0	2.09810	1.16662
RE	21.7	2.09796	1.16491	MN	8.7	ND	ND
RE	18.4	2.09966	1.16455	MN	7.8	ND	ND
RE	50.6	2.10021	1.16197	MN	6.3	ND	ND
				MN	7.1	ND	ND

#Sample code are as follow: SOR, Lambrusco di Sorbara; GRA, Lambrusco Grasparossa di Castelvetro; SAL, Lambrusco Salamino di S. Croce; MN, Lambrusco Mantova; RE, Lambrusco Reggio Emilia; ND, not determined. §The RSD% and uncertainty values, associated to the reported data, were evaluated by repeated and replicated measurements performed on the soil control sample.

Table S5. Operating parameters used to measure the isotopic ratio with the Neptune MC-ICP/MS spectrometer.

Parameter	Setup value
Rf power	1245 W
Gas flow rate	Sample gas: 0.992 L min^{-1}<br>Auxiliary gas: 0.85 L min^{-1}<br>Cooling gas: 15 L min^{-1}
Sample/Skimmer cone	Ni
Spray chamber	Cyclonic + Scott type or APEX-IR
Nebulizer	PFA micro flow self-aspirating
Faraday Cup configuration - Amplificators	L3 – ^{202}Hg - 10^{12}<br>L2 – ^{203}Tl - 10^{12}<br>L1 – ^{204}Pb - 10^{12}<br>C – ^{205}Tl - 10^{11}<br>H1 – ^{206}Pb - 10^{11}<br>H2 – ^{207}Pb - 10^{11}<br>H3 – ^{208}Pb - 10^{11}
Control Cup peak center	C – ^{205}Tl
Mass analyzer pressure	$<10^{-8}$ bar
Background/baseline determination	4% HNO_3 (*v/v*) or 3M HCl
Sample uptake rate	100 μL min^{-1}
Uptake time	160 s
Wash time	100 s
Idle time	3 s
Number of blocks	1
Integration time	4.194 s
Number of cycles	100
Time of measurement for each sample	12 min
Sensibility	≥20 V/ppm for ^{208}Pb
APEX-IR	
Heater temperature	100 °C
Chiller temperature	2 °C
Sensibility	80 V/ppm for ^{208}Pb

Table S6. Averaged lead I.R. values (n = 18) obtained with different mass bias correction methods determined on the control soil sample and respective uncertainties, u = 2s.

		Control sample	
**Corr. method**	**I.R.**	$^{208}Pb/^{206}Pb$	$^{207}Pb/^{206}Pb$
(a) C_{factor}		2.07537 ± 0.00067	0.83976 ± 0.00022
(b) $f_{Pb} = f_{Tl}$		2.07450 ± 0.00037	0.83987 ± 0.00022
(c) $f_{Pb} \neq f_{Tl}$		2.07474 ± 0.00029	0.83992 ± 0.00016

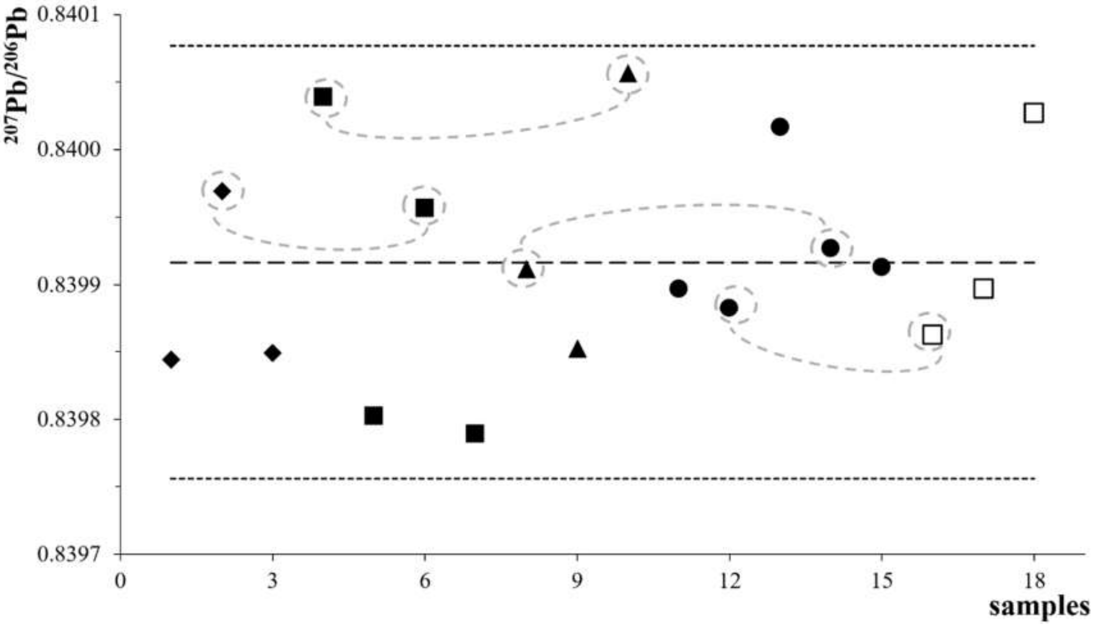

Figure S1. $^{207}Pb/^{206}Pb$ isotopic ratio data for the separated control soil samples (N = 18) obtained with the internal correction considering $f_{Pb} \neq f_{Tl}$. (◆) data of 14/06/2016, (■) data of 04/07/2016, (▲) data of 13/09/2016, (●) data of 26/09/2016, (□) data of 17/01/2017, (- -) mean value. Dotted upper and bottom lines show the uncertainty value interval associated to the mean value of the data: 0.83992 ± 0.00016. Linked values correspond to the same control sample measured in two consecutive working sessions.
